# Deficiency of the bone mineralization inhibitor NPP1 protects mice against obesity and diabetes

**DOI:** 10.1242/dmm.017905

**Published:** 2014-10-31

**Authors:** Carmen Huesa, Dongxing Zhu, James D. Glover, Mathieu Ferron, Gerard Karsenty, Elspeth M. Milne, José Luis Millan, S. Faisal Ahmed, Colin Farquharson, Nicholas M. Morton, Vicky E. MacRae

**Affiliations:** 1 The Roslin Institute and Royal (Dick) School of Veterinary Studies, University of Edinburgh, Easter Bush, Roslin, Midlothian, Edinburgh, EH25 9RG, UK.; 2 Integrative and Molecular Physiology Research Unit Institut de Recherches Cliniques de Montréal (IRCM), 110 Avenue des Pins Ouest – Laboratory 2750, Montréal, QC H2W 1R7, Canada.; 3 Department of Developmental Genetics, Columbia University, NY 10032, USA.; 4 Sanford Children’s Health Research Center, Sanford-Burnham Medical Research Institute, La Jolla, CA 92037, USA.; 5 Developmental Medicine, University of Glasgow, Glasgow, G12 8QQ, UK.; 6 Centre for Cardiovascular Science, Queen’s Medical Research Institute, University of Edinburgh, Edinburgh, EH16 4TJ, UK.

**Keywords:** NPP1, Mineralization, Obesity, Diabetes

## Abstract

The emergence of bone as an endocrine regulator has prompted a re-evaluation of the role of bone mineralization factors in the development of metabolic disease. Ectonucleotide pyrophosphatase/phosphodiesterase-1 (NPP1) controls bone mineralization through the generation of pyrophosphate, and levels of NPP1 are elevated both in dermal fibroblast cultures and muscle of individuals with insulin resistance. We investigated the metabolic phenotype associated with impaired bone metabolism in mice lacking the gene that encodes NPP1 (*Enpp1^−/−^* mice). *Enpp1*^−/−^ mice exhibited mildly improved glucose homeostasis on a normal diet but showed a pronounced resistance to obesity and insulin resistance in response to chronic high-fat feeding. *Enpp1*^−/−^ mice had increased levels of the insulin-sensitizing bone-derived hormone osteocalcin but unchanged insulin signalling within osteoblasts. A fuller understanding of the pathways of NPP1 could inform the development of novel therapeutic strategies for treating insulin resistance.

## INTRODUCTION

Bone remodelling is a highly conserved and regulated process that controls bone homeostasis and maintains skeletal structural integrity. This vital function is characterized by alternating phases of bone resorption by osteoclasts and bone formation by osteoblasts, and has a high energetic cost (Ducy et al., 2000). It has recently been proposed that insulin signalling mediates communication between bone remodelling and metabolic control (Ferron et al., 2010a; Fulzele et al., 2007; Lee et al., 2007). Osteocalcin, a secreted protein that is specifically expressed in osteoblasts, is essential for this function of the skeleton. Hormonally active osteocalcin (in an under-or un-carboxylated form) acts to increase β-cell proliferation, insulin secretion, peripheral insulin sensitivity and energy expenditure (Ferron et al., 2008; Fulzele et al., 2010). This raises the possibility that other factors controlling bone remodelling impact upon metabolic homeostasis.

Ectonucleotide pyrophosphatase/phosphodiesterase-1 (NPP1), also known as plasma cell membrane glycoprotein 1 (PC-1), is the founding member of the NPP family – a family consisting of seven isozymes with a structurally related catalytic domain. The NPPs hydrolyze phosphodiester or pyrophosphate bonds in various substrates, including nucleoside triphosphates, lysophospholipids and choline phosphate esters (Bollen et al., 2000; Stefan et al., 2005; Zimmermann et al., 2012). NPP1 is a glycoprotein that forms disulphide-bonded homodimers in both the plasma membrane and mineral-depositing matrix vesicles of osteoblasts and chondrocytes (Hessle et al., 2002; Johnson et al., 2001; Terkeltaub, 2006; Vaingankar et al., 2004). NPP1 hydrolyzes extracellular nucleotides into inorganic pyrophosphate (PPi), a potent inhibitor of hydroxyapatite (HA) crystal formation in mineralization-competent tissues (Terkeltaub, 2001). Mice lacking NPP1 (*Enpp1^−/−^*) have severe mineralization defects, which are associated with abnormally low PP_i_ levels (Anderson et al., 2005; Johnson et al., 2003; Sali et al., 1999). Phenotypic features of *Enpp1^−/−^* mice include marked alterations in the mineralization of long bones and calvaria, and pathologic calcification of the perispinal soft tissue and medial arterial layer (Mackenzie et al., 2012a; Mackenzie et al., 2012b; Sali et al., 1999).

Impaired insulin action (insulin resistance) is a key risk factor for type 2 diabetes (DeFronzo, 1988). NPP1 negatively modulates insulin receptor signalling and has been proposed as a pathogenic factor predisposing to insulin resistance (Goldfine et al., 2008; Prudente et al., 2009). *ENPP1* is overexpressed in skeletal muscle, adipose tissue, fibroblasts and lymphocytes of insulin-resistant individuals (Frittitta et al., 1997; Frittitta et al., 1998; Goldfine et al., 2008; Stentz and Kitabchi, 2007; Teno et al., 1999). Additionally, overexpression of NPP1 in cultured cells inhibits insulin receptor autophosphorylation and downstream signalling (Grupe et al., 1995). Further studies have shown that NPP1 binds to insulin receptor and inhibits the insulin-induced conformational changes that lead to insulin receptor autophosphorylation and tyrosine kinase activation (Maddux et al., 1995; Maddux and Goldfine, 2000). Studies on the NPP1 Lys121Gln (K121Q) polymorphism (Pizzuti et al., 1999), a putative genetic determinant of human insulin resistance, lent further support to NPP1 having a role in the etiology of human insulin resistance. *In vitro* studies have provided evidence for the increased susceptibility to insulin resistance of the Gln121 variant (Di Paola et al., 2011), with a Lys121Gln meta-analysis conducted on a European population showing a modest increase of the Gln allele in those at risk of type 2 diabetes (McAteer et al., 2008).

Despite the recognized importance of NPP1 in the control of bone mineralization, its contribution to the regulation of glucose metabolism is less clear. Given that elevated NPP1 is associated with insulin resistance (Goldfine et al., 2008; Prudente et al., 2009), we hypothesized that *ENPP1* gene deletion would promote improved glucose homeostasis in the context of obesity-associated diabetes. To test this we challenged *Enpp1^−/−^* mice with chronic exposure to a high-fat diet (HFD).

**TRANSLATIONAL IMPACT**

**Clinical issue**

More than half a billion individuals worldwide suffer from obesity, which is often associated with other metabolic diseases, such as type 2 diabetes. The emergence of bone as an endocrine regulator has led to the re-evaluation of the role of bone mineralization factors in the development of metabolic disease. Despite the recognized importance of ectonucleotide pyrophosphatase/phosphodiesterase-1 (NPP1) in the regulation of bone mineralization, its contribution to obesity and type 2 diabetes remains less clear.

**Results**

This research investigates the metabolic phenotype associated with impaired bone metabolism in mice that lack the gene encoding NPP1 (*Enpp1^−/−^* mice). New evidence is provided showing that *Enpp1^−/−^* mice have a pronounced resistance to obesity and to the development of insulin resistance in response to chronic high-fat feeding. Furthermore, the authors show that *Enpp1^−/−^* mice have increased levels of the insulin-sensitizing bone-derived hormone osteocalcin, although insulin signalling remained unchanged within osteoblasts (cells involved in bone formation) in these mice.

**Implications and future directions**

These findings shed light on the important role of NPP1 in the development of obesity and type 2 diabetes, and provide new insights into the mechanism by which this protein regulates insulin sensitivity. A deeper understanding of the pathways that are regulated by NPP1 might advance the development of novel therapeutic strategies for treating insulin resistance.

## RESULTS

### *Enpp1^−/−^* mice show unaltered fat mass on control diet

**Fig. 1.**
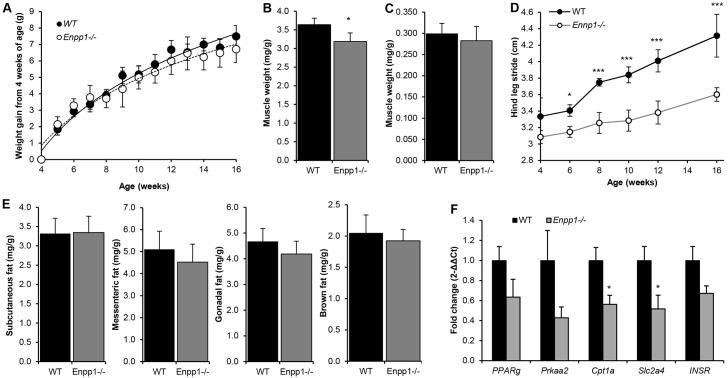
***Enpp1^−/−^* mice have significant muscle reduction but not significant changes in fat mass.** (A) Body weight (g) growth curves for *Enpp1^−/−^* and WT mice (*n*=6). (B,C) *Enpp1^−/−^* mice show (B) reduced quadratus femoris and (C) unaltered soleus muscle weight relative to total body weight (mg/g), compared with WT controls (*n*=6). (D) Walking gait stride length of *Enpp1^−/−^* and WT mice (*n*=4). (E) Subcutaneous, mesenteric, gonadal and brown fat pad weight relative to total body weight (mg/g) compared with WT controls (*n*=6). (F) Relative expression of WAT markers and *Insr* in the subcutaneous fat pad. Mice were reared under control dietary conditions and were 18 weeks of age at the time of dissection. Results are presented as mean±s.e.m. *P<0.05, ***P<0.001.

There was no significant difference in body weight gain from 4 weeks of age between wild-type (WT) and *Enpp1*^−/−^ mice (Fig. 1A), yet a significant reduction in quadratus femoris muscle mass was observed from 4 weeks of age in *Enpp1*^−/−^ mice (12%; *P*<0.05; Fig. 1B) with no differences in soleus muscle (Fig. 1C). The loss of muscle mass in the quadratus femoris, a key target of exercise loading, is a likely consequence of the debilitating arthritis that the *Enpp1*^−/−^ mice exhibit (Filippin et al., 2013). No effect of genotype on food intake was noted. To assess the onset and severity of arthritis, stride length was assessed using walking gait analysis. From 6 weeks of age, the stride length of the *Enpp1*^−/−^ mice was significantly reduced (*P*<0.05), as was the progressive increase in stride length with advancing age (Fig. 1D). No differences in mRNA expression of muscle genes such as *Mstn*, *Fbxo32* or *Fndc5* were noted in these muscles (data not shown).

There were no differences in white (gonadal, subcutaneous and mesenteric) or brown fat mass (Fig. 1E), and no changes in mRNA levels of key genes for adipogenesis (*Pparg*), lipolysis (*Prkaa20*), mitochondrial metabolism (*Cpt1a*) or glucose transport (*Slc2a4*) in gonadal or brown fat mass (data not shown). However, *Cpt1a* and *Slc2a4* mRNA expression in the subcutaneous fat mass was significantly reduced in *Enpp1*^−/−^ mice compared with WT (50%; *P*<0.05; Fig. 1F).

### Chronic deficiency of NPP1 in mice induces insulin sensitization on control diet

**Fig. 2.**
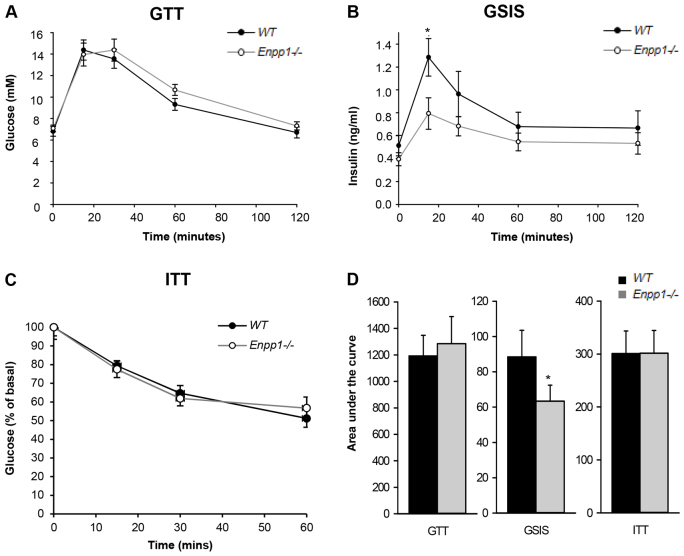
***Enpp1^−/−^* mice exhibit insulin sensitization.** (A) Glucose tolerance test (GTT). (B) Glucose-stimulated insulin secretion (GSIS). (C) Insulin tolerance test (ITT) represented as percentage (%) of basal glucose. (D) Metabolic tests analyzed as area under the curve. Mice were reared under control dietary conditions to 16 weeks of age. Results are presented as mean±s.e.m. (*n*=8). *P<0.05.

**Fig. 3.**
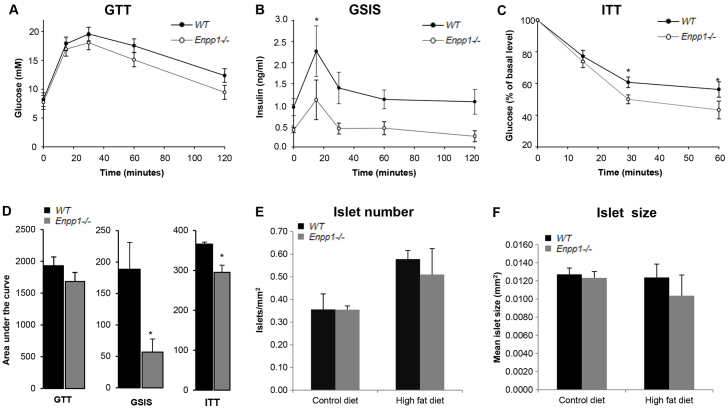
***Enpp1^−/−^* mice show improved insulin tolerance in response to a chronic HFD challenge.** (A) Glucose tolerance test (GTT). (B) Glucose-stimulated insulin secretion (GSIS). (C) Insulin tolerance test (ITT) represented as percentage (%) of basal glucose. Mice were reared under high-fat dietary conditions and were 16 weeks of age. Results are presented as mean±s.e.m. (*n*=7). *P<0.05. (D) Metabolic tests analyzed as area under the curve. (E) Number of islets. (F) Size of islets. Results are presented as mean±s.e.m. *P<0.05.

Owing to the recognized inhibitory activity of NPP1 on the insulin receptor (Maddux and Goldfine, 2000), we tested whether global deletion of *Enpp1* would translate into changes in whole-body glucose metabolism. Adult *Enpp1^−/−^* mice showed normal glucose tolerance tests (GTTs) (Fig. 2A,D) but with a lower glucose-stimulated insulin secretion (GSIS) peak across the GTT, indicating insulin sensitization (Fig. 2B,D). Adult *Enpp1^−/−^* mice also showed normal insulin tolerance tests (ITTs) (Fig. 2C,D) and there were no differences in the size or number of insulin-secreting islets in the pancreas of *Enpp1^−/−^* mice compared with WT (Fig. 3E,F).

### *Enpp1^−/−^* mice show obesity resistance and improved insulin tolerance in response to chronic HFD challenge

**Fig. 4.**
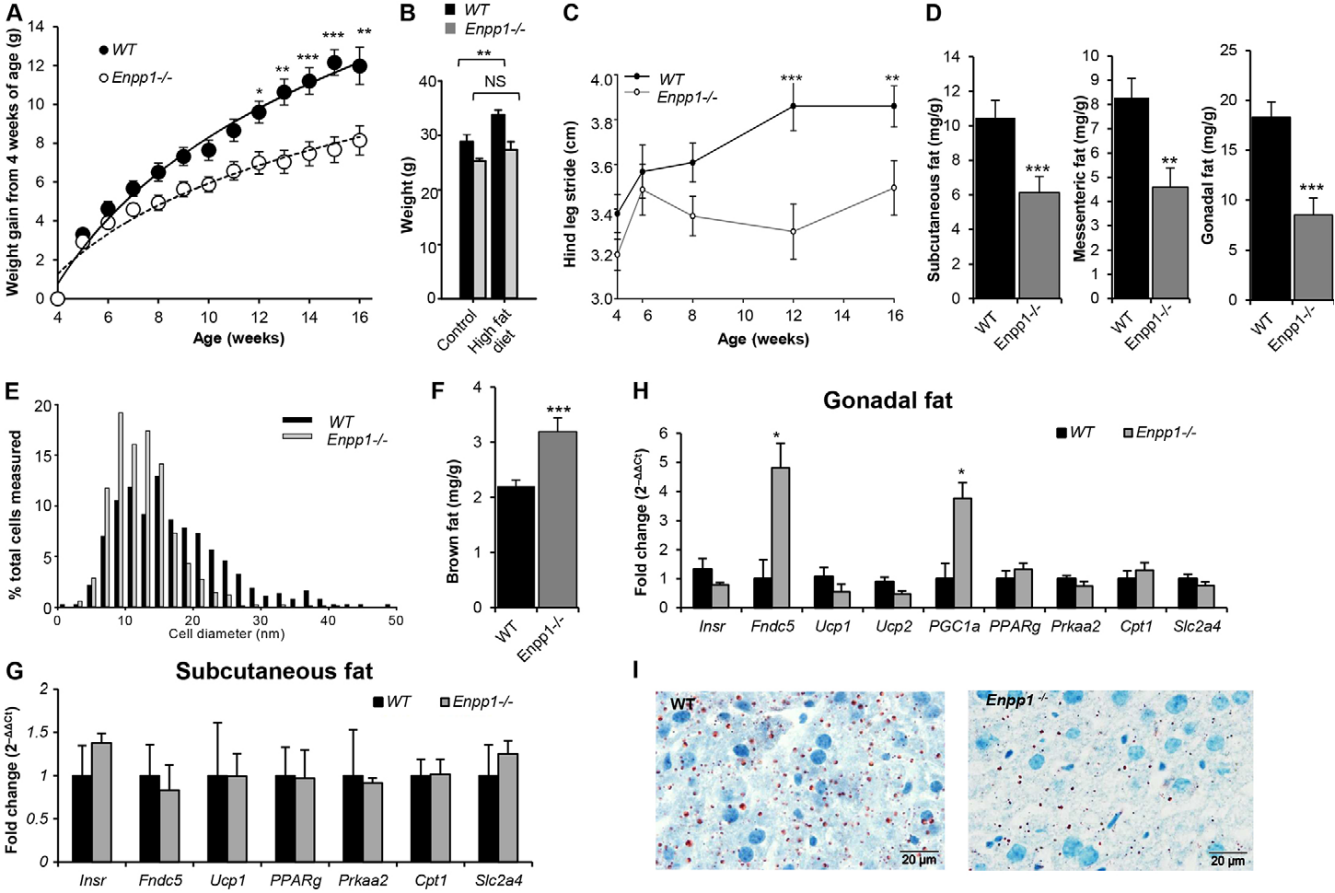
***Enpp1^−/−^* mice show pronounced obesity resistance in response to a chronic HFD challenge.** *Enpp1^−/−^* and WT mice were reared on a HFD from 4 weeks of age. (A) Body weight (g) growth curves (*n*=16). (B) Body weight (g) at 18 weeks of age (*n*=8). (C) Walking gait stride length in WT and *Enpp1^−/−^* mice (*n*=4). (D) WAT weight analysis as a ratio to total body weight (mg/g) (*n*=8). (E) Maximum gonadal fat cell diameter (*n*=4). (F) BAT weight analysis as a ratio to total body weight (*n*=8). (G,H) Gene expression of WAT and BAT markers and *Insr* in (G) subcutaneous and (H) gonadal fat pad. (I) Histological Oil Red O staining of lipid deposits in cryosections of the liver. Mice were analyzed at 18 weeks of age after 14 weeks under high-fat dietary conditions. Results are presented as mean±s.e.m. *P<0.05, **P<0.01, ***P<0.001, NS: non-significant.

Chronic high-fat feeding caused weight gain in WT mice, but *Enpp1^−/−^* mice showed resistance to this (Fig. 4A,B). Of note, quadratus femoris mass increased with a HFD in the *Enpp1*^−/−^ mice (40% increase compared with control diet; *P*<0.05), whereas walking gait, as denoted by stride length, remained significantly reduced (*P*<0.05) (Fig. 4C).

*Enpp1^−/−^* mice showed reduced white adipose tissue (WAT) mass (*P*<0.01; Fig. 4D) and adipocyte cell diameter (Fig. 4E) compared with WT. Conversely, a marked increase in brown fat mass (37%; *P*<0.001; Fig. 4F) was noted. The gonadal fat pad showed a significant increase in *PGC1α* and *Fndc5* expression (Fig. 4H), which are indicative of increased mitochondrial biogenesis. However, there was no change in *Ucp1* mRNA levels (Fig. 4H) and hence no evidence for increased canonical thermogenesis or white fat browning as a mechanism underlying the reduced adiposity and improved metabolic profiles. In WAT, expression of white fat markers (e.g. *Cpt1*, *Prkaa2*) remained unaltered between WT and *Enpp1^−/−^* mice with a HFD (Fig. 4G,H). Moreover, microvesicular fat in the liver, as detected by Oil Red O staining, was notably reduced in *Enpp1*^−/−^ mice in comparison to WT littermates (Fig. 4I). No gross effect of HFD challenge was seen on kidney, brain, cardiovascular or muscle tissue architecture in either genotype.

Glucose tolerance was comparable between genotypes (Fig. 3A,D). However, the insulin level (GSIS) across the GTT was lower in *Enpp1^−/−^* mice, indicating potential insulin sensitization (Fig. 3B,D). Improved *Enpp1^−/−^* insulin sensitivity was confirmed by an ITT (Fig. 3C,D). Compared with wild-type control mice, no significant differences in the size or number of insulin-secreting islets were observed in the pancreas of *Enpp1^−/−^* mice (Fig. 3E,F).

### Do osteoblasts regulate insulin sensitivity in *Enpp1^−/−^* mice?

**Fig. 5.**
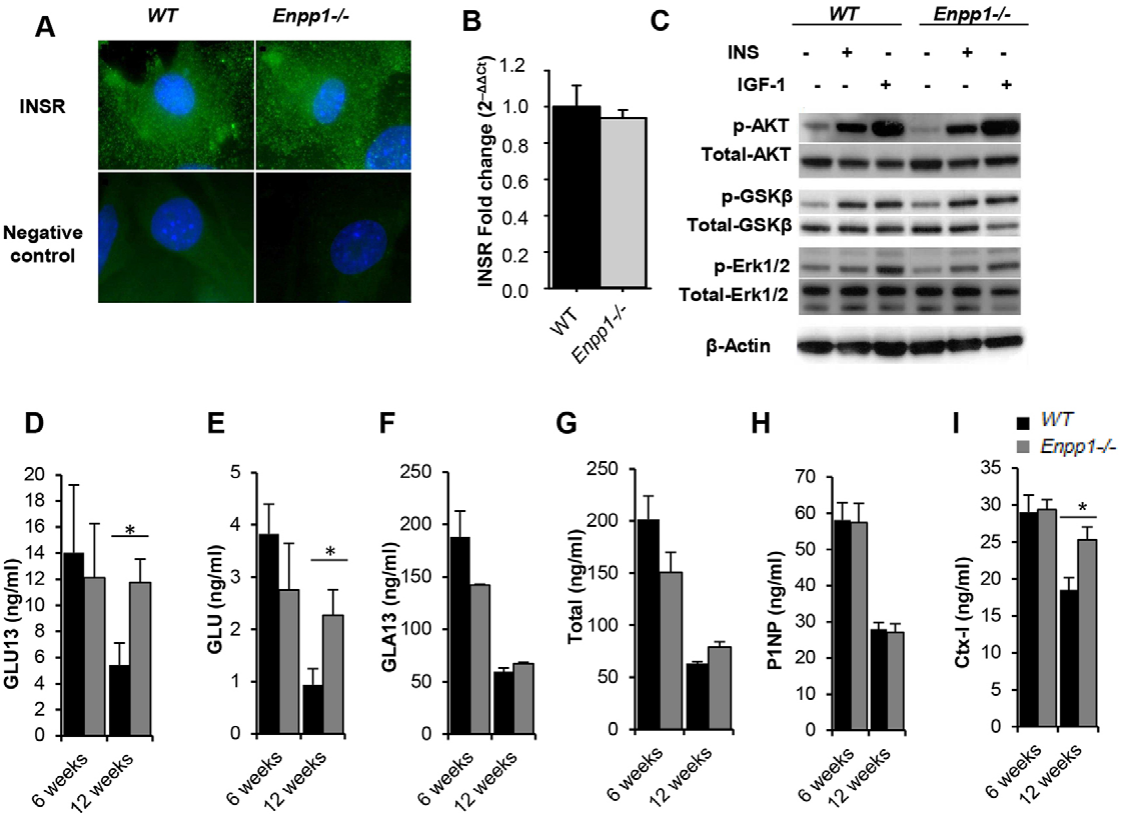
**Metabolic effects in *Enpp1^−/−^* mice are independent of bone insulin signalling but associated with altered osteocalcin carboxylation status.** (A) Immunofluorescent staining of the insulin receptor (green) on the surface of WT and *Enpp1^−/−^* neonatal calvarial primary osteoblast cultures. Nuclei are shown in blue. (B) Relative mRNA expression levels of *Insr* in calvarial osteoblast cultures (*n*=6). (C) Representative immunoblots demonstrating the effects of insulin (INS; 10 nM) and IGF-1 (10 nM) on the phosphorylation of Akt, glycogen synthase kinase-3 beta (GSK-3β) and Erk1/2 in calvarial osteoblast cultures (*n*≥3). (D-I) Measurement of serum (D) undercarboxylated osteocalcin, (E) uncarboxylated osteocalcin, (F) carboxylated osteocalcin, (G) total osteocalcin, (H) bone-formation marker P1NP and (I) bone-resorption marker CTx-I in 6- and 12-week-old mice. Results are presented as mean±s.e.m. (*n*=6). *P<0.05.

Given that NPP1 is a negative regulator of insulin signalling, we investigated whether osteoblast insulin signalling might link NPP1 deficiency and improved whole-body glucose homeostasis. Initially, we considered the distribution (Fig. 5A) and mRNA expression (Fig. 5B) of the insulin receptor in confluent monolayers of primary calvarial osteoblasts, with no effect of genotype observed. Furthermore there was no effect of NPP1 deletion on insulin-stimulated Akt, Erk1/2 or GSK3β phosphorylation between *Enpp1^−/−^* and WT primary calvarial osteoblasts cultured *in vitro* (Fig. 5C). However, at 12 weeks of age, *Enpp1^−/−^* mice exhibited increased concentrations of the osteoblast-derived insulin-sensitizing hormone undercarboxylated (GLU13) (119%, *P*<0.05; Fig. 5D) and uncarboxylated (GLU) (156%, *P*<0.05; Fig. 5E) osteocalcin, whereas carboxylated (GLA13) and total osteocalcin levels were comparable to WT (Fig. 5F,G). Additionally, the degree of osteoblast and osteoclast activity was assessed by ELISA analysis of serum from *Enpp1^−/−^* and WT mice. No changes in the bone-formation marker P1NP were noted (Fig. 5H), whereas a significant increase in the bone-resorption marker CTx was observed in *Enpp1^−/−^* mice compared with WT controls (*P*<0.05; Fig. 5I). Together, these data suggest that the increase in undercarboxylated osteocalcin detected in *Enpp1*^−/−^ mice as a result of increased bone resorption might induce the phenotype of increased insulin-sensitivity observed.

### Chronic HFD challenge induces brittle-bone formation in WT mice and exacerbates the bone phenotype of *Enpp1^−/−^* mice

Given the detrimental effects of obesity on bone (Leslie et al., 2012), we next considered the effects of chronic high-fat challenge on the bone physiology of *Enpp1*^−/−^ mice. Following a chronic HFD challenge, trabecular bone in WT mice showed a significantly altered microarchitecture resulting in thinner and more disorganized trabeculi, compared with mice reared on a control diet (Table 1). Cortical bone in WT mice fed a HFD showed a significantly increased bone mineral density (BMD) compared with mice reared on a control diet (*P*<0.01). However, cortical thickness was unchanged by the HFD, resulting in a more brittle bone as determined by the significantly decreased work to fracture (*P*<0.05) and post-failure work (*P*<0.05) observed in the three-point bending analysis (work is defined as the necessary force to cause a displacement and is calculated as the area under the curve) (Table 1).

**Table 1.**
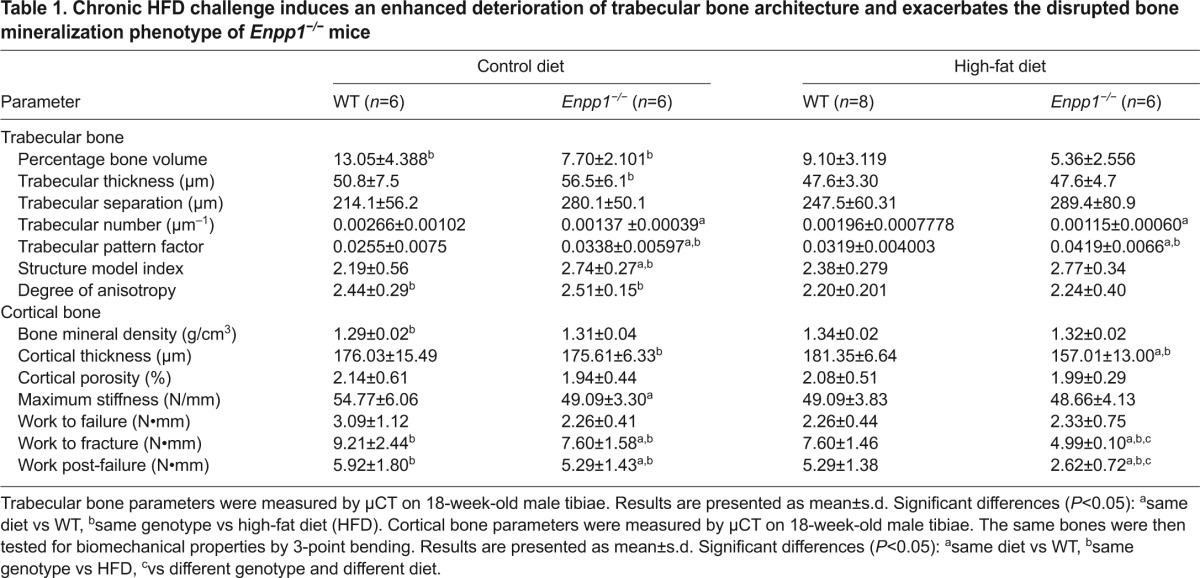
Chronic HFD challenge induces an enhanced deterioration of trabecular bone architecture and exacerbates the disrupted bone mineralization phenotype of *Enpp1^−/−^* mice

**Fig. 6.**
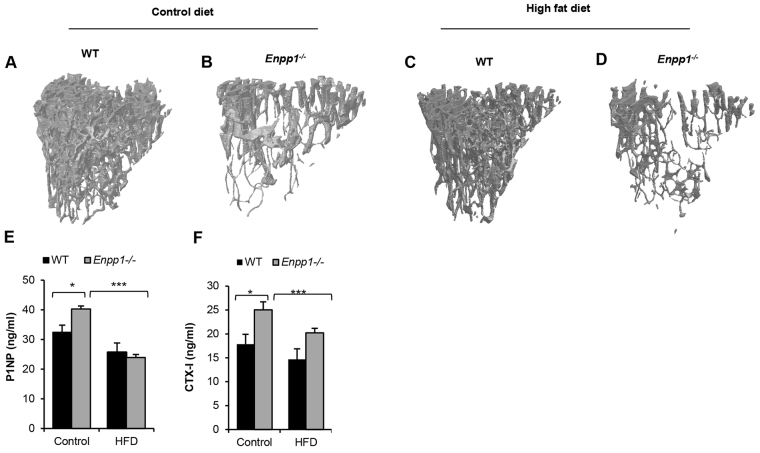
**HFD induces a deterioration of trabecular bone architecture.** (A-D) Representative images of trabecular bone from 18-week-old mice tibiae. (A) WT control diet. (B) *Enpp1^−/−^* control diet. (C) WT HFD. (D) *Enpp1^−/−^* HFD. (E) Bone-formation marker P1NP and (F) bone-resorption marker CTx-I in 18-week-old WT and *Enpp1^−/−^* mice. Results are presented as mean±s.e.m. (*n*=6). *P<0.05, ***P<0.001.

*Enpp1*^−/−^ mice displayed a significant deterioration of the already compromised trabecular architecture following a chronic HFD challenge, as exemplified by the significant loss of connectivity indicated by the trabecular pattern factor, the loss of trabecular bone as measured by percentage of bone volume (BV/TV) and the reduced trabecular organization when compared with control-diet mice (Table 1 and Fig. 6A–D). The tibiae of *Enpp1*^−/−^ mice displayed a significantly reduced cortical thickness (10%; *P*<0.01). This also translated into a weaker, more brittle bone as measured by three-point bending parameters whereby the work to fracture and post-failure was significantly reduced by 34% and 50%, respectively (*P*<0.05; Table 1). A HFD challenge also induced a significant decrease in bone modelling in *Enpp1*^−/−^ mice, as indicated by the significant reduction in P1NP as a marker of bone formation (38%; *P*<0.001; Fig. 6E) and CTx as a marker of bone resorption (20%; *P*<0.001; Fig. 6F). These unique data highlight that a chronic high-fat challenge has crucial effects on the severity of the *Enpp1*^−/−^ bone phenotype.

## DISCUSSION

The role of NPP1 in controlling the pace of bone mineralization has been firmly established through the study of *in vivo* mouse models. This enzyme is crucial for the regulation of bone mineralization through the generation of PPi from nucleotide precursors. *Enpp1*^−/−^ mice display a distinct phenotype, including reduced long-bone and increased calvarial mineralization, and pathologic soft-tissue calcification (Mackenzie et al., 2012a; Mackenzie et al., 2012b; Sali et al., 1999). The necessity of NPP1 for physiological skeletal mineralization combined with the considerable evidence linking NPP1 expression with insulin resistance (Bacci et al., 2007; Baratta et al., 2008; Chandalia et al., 2012; González-Sánchez et al., 2008; Harmey et al., 2004) led us to investigate whether NPP1 has a functional role in bone as a newly identified regulator of energy metabolism.

In the present study we have examined the full impact of chronic suppression of NPP1 and provide new evidence demonstrating that ablation of the bone mineralization inhibitor NPP1 protects against insulin resistance, obesity and diabetes. *Enpp1*^−/−^ mice exhibited insulin sensitization and, in response to a chronic HFD challenge, displayed improved insulin tolerance and pronounced obesity resistance. These novel data extend recent *in vitro* studies where the suppression of NPP1 expression in liver, skeletal muscle and pancreatic β-cells improved insulin sensitivity (Di Paola et al., 2011). The observation of enhanced insulin sensitivity following adenoviral knockdown of liver NPP1 expression in a mouse model of diabetes (db/db; *Lepr^−/−^*) further supports our findings (Zhou et al., 2009). In comparison with db/db mice treated with a control virus, db/db mice treated with the *Enpp1*-shRNA adenovirus had 80% lower hepatic *Enpp1* mRNA levels, 25% lower fasting plasma glucose and significantly improved glucose tolerance. This crucial paper (Zhou et al., 2009) highlights the bone-independent effects of NPP1 and, together with our findings, supports the proposition that NPP1 inhibition is a potential therapeutic approach for the treatment of type 2 diabetes.

The present study provides the first demonstration of an association between osteocalcin carboxylation status and NPP1. *Enpp1*^−/−^ mice presented with increased levels of undercarboxylated osteocalcin and the bone-resorption marker CTx. These data support recent findings indicating that insulin signalling in osteoblasts favours bone resorption by osteoclasts, with decarboxylation of osteocalcin occurring in resorption lacunae, resulting in increased circulating undercarboxylated osteocalcin and a positive feedback loop on insulin secretion (Ferron et al., 2010a; Lacombe et al., 2013). However, our studies undertaken in calvarial osteoblast cultures did not reveal a role for NPP1 as a modulator of insulin signalling in osteoblasts *in vitro*, suggesting that this model might not fully reflect osteoblast activity *in vivo*. Further studies involving the specific knockout of the insulin receptor in *Enpp1*^−/−^ osteoblasts are therefore required to categorically confirm these data. Given the recent important observation that osteoclasts regulate energy metabolism (Lacombe et al., 2013), it is also vital to determine whether *Enpp1*^−/−^ osteoclasts show any functional defects.

The distinct phenotype of the *Enpp1*^−/−^ mouse might also directly influence the metabolic parameters observed in the present study. *Enpp1*^−/−^ mice showed reduced body weight, decreased stride length and significant muscle reduction. These data are highly compatible with the observed osteoarthritic phenotype (Mackenzie et al., 2012a; Sali et al., 1999), which might modulate the insulin sensitization induced by NPP1 loss. Indeed, metabolic osteoarthritis (OA) has now been characterized as a subtype of OA, and has been highlighted as a new facet of the definition of metabolic syndrome, supported by its strong associations and shared mechanisms with other metabolic syndrome components (Zhuo et al., 2012). Further research, however, is needed to define the reciprocal influence of OA on the currently accepted components of metabolic syndrome, and the putative role of NPP1.

The insulin-sensitized phenotype observed in the *Enpp1*^−/−^ mice might be a direct consequence of NPP1 ablation in insulin-sensitive tissues such as liver, adipose and skeletal muscle. Overexpression of NPP1 in liver and muscle induces insulin resistance and hyperglycemia (Maddux et al., 2006). Moreover, overexpression of NPP1 in adipocytes leads to adipose insulin resistance, reduction in fat-cell lipid storage and systemic insulin resistance and glucose intolerance akin to the effects of lipodystrophy (Pan et al., 2011). Our data support a beneficial effect of NPP1 deficiency being manifested potentially through improved insulin sensitivity in liver, muscle and adipose. However, one key difference in the *Enpp1*^−/−^ mice is that insulin sensitization is associated with pronounced reduction in adipose tissue lipid storage without ectopic lipid accumulation in other organs. This suggests an overarching beneficial effect of *Enpp1* deficiency on energy expenditure that likely involves other tissues. This could include increased muscle mitochondrial biogenesis through PGC1-α, a key regulator of energy metabolism (Ferron et al., 2012) or effects of increased bone turnover. Tissue-specific ablation of NPP1 is therefore required in future work to pinpoint the individual tissue(s) driving the metabolic phenotype of the *Enpp1*^−/−^ mouse. Furthermore, the association of the NPP1 K121Q polymorphism with insulin resistance in several human populations (Baratta et al., 2008; Stolerman et al., 2008) highlights the need for further studies specifically investigating the impact of this variant of NPP1 on metabolism. One of the premises of targeting NPP1 as treatment for type-2-diabetes-induced insulin resistance is that the inhibition of insulin receptor and NPP1 interactions happens independently of its pyrophosphatase/phosphodiesterase activity, although the exact mechanism is still unknown (Chin et al., 2009).

Obesity is linked to normal or higher BMD accompanied by a paradoxical increase in fracture risk (Leslie et al., 2012). Our studies mimic this clinical setting of an obesity-induced bone phenotype in the WT mouse, where the bone is more brittle despite a higher BMD. Furthermore, our data show that a HFD challenge induces a decrease in bone modelling that results in an enhanced deterioration of bone architecture and mechanical properties; this is even more pronounced in *Enpp1*^−/−^ mice. This latter observation might reflect the abnormally low levels of extracellular PPi observed in these mice, which would result in insufficient PPi substrate for tissue nonspecific alkaline phosphatase (TNAP) to generate Pi for normal mineral formation, as previously discussed (Anderson et al., 2005). Furthermore, an accumulation of nucleotide triphosphates due to lack of hydrolysis by NPP1 (Prosdocimo et al., 2009) could have a downstream effect on bone remodelling through purinergic signalling (Orriss et al., 2010). No differences in cortical porosity were seen between either genotype or diet; however, it was not possible to distinguish between vascularity, osteocyte lacunae and areas of non-mineralized matrix at the resolution examined. Further investigations specifically examining these individual components using nano-CT would therefore be of great interest.

**Fig. 7.**
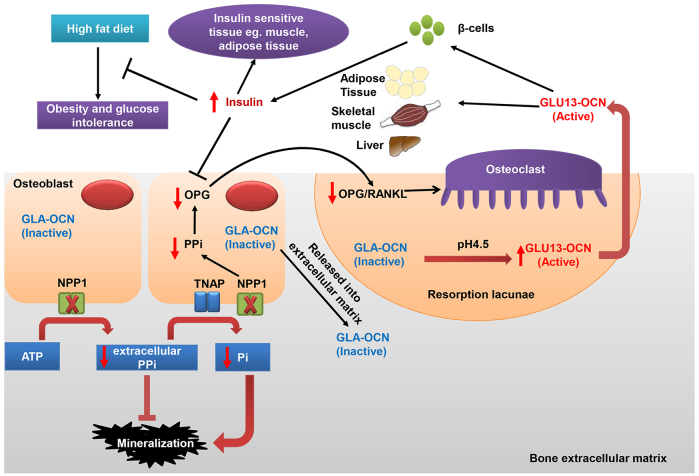
**Schematic representation of the effects of NPP1 on bone mineralization, obesity and glucose intolerance.** NPP1 hydrolyses ATP to generate pyrophosphate (PPi), which is a key inhibitor of mineralization. Global deletion of *Enpp1* results in decreased PPi levels, leading to ectopic calcification of blood vessels and cartilage. However, in long bones such as tibiae, *Enpp1* ablation leads to insufficient PPi substrate for TNAP to generate Pi for normal mineral formation. On the other hand, decreased PPi might reduce the ratio of OPG to RANKL, resulting in increased bone resorption by osteoclasts (Ribeiro et al., 2014). The acidic pH (4.5) in resorption lacunae decarboxylates osteocalcin (GLA-OCN) stored in the bone extracellular matrix to generate undercarboxylated active osteocalcin (GLU13-OCN), which stimulates insulin secretion by the β-cells of the pancreatic islets and promotes insulin sensitivity (Ferron and Lacombe, 2014). Increased insulin binds to the insulin receptor in osteoblasts and inhibits the expression of OPG, driving osteoclastic bone resorption and the further release of GLU13-OCN. Increased insulin secretion protects HFD-induced obesity and glucose intolerance, and impacts on insulin-sensitive tissues, such as muscle and adipose. Additionally, GLU13-OCN can directly stimulate energy consumption in skeletal muscle and adipose tissue and might decrease lipid accumulation in the liver (Rosen and Motyl, 2010).

In conclusion, our data adds to the body of evidence (Goldfine et al., 2008; Prudente et al., 2009) supporting NPP1 deficiency as being protective against insulin resistance. We demonstrate for the first time that ablation of NPP1 alters osteocalcin carboxylation status, while protecting against obesity and diabetes (Fig. 7). A fuller understanding of the pathways of NPP1 could advance the development of novel therapeutic strategies for treating insulin resistance.

## MATERIALS AND METHODS

### Animal model

The generation and characterization of germline *Enpp1^−/−^* mice have been reported previously (Mackenzie et al., 2012a; Sali et al., 1999). Heterozygote breeders were used to generate distinct litters. Offspring carrying the mutant *Enpp1* gene were identified by PCR and performed by Genetyper (Genetyper, NY, USA). Male mice were fed a HFD (58% fat; DBM Scotland, Broxburn, UK) or control diet (6.2% fat; Harlan Laboratories, Indianapolis, IN, USA) from 4 weeks of age. *Ad libitum* food consumption was monitored for 6 days. Progression of arthritis was assessed with a non-automated ‘cat-walk’ walking gait test, which measured the distance between the central pads of two alternating hind footprints (Parvathy and Masocha, 2013). All animal experiments were approved by The Roslin Institute’s Animal Users Committee and the animals were maintained in accordance with UK Home Office guidelines for the care and use of laboratory animals.

### Glucose and insulin tolerance tests

Juvenile and adult (6- and 16-week-old, respectively) male mice were fasted for 4 hours and administered 2 mg of D-glucose (Sigma, Poole, UK) per g of body weight by gavage. 16-week-old male mice were fasted for 4 hours and administered 0.5 (control diet) or 0.75 (HFD) mU of insulin (Actrapid, NovoNordisk, Bagsvaerd, Denmark) per g of body weight, whereas 6-week-old male mice were administered 0.375 mU of insulin per g of body weight intraperitoneally (i.p.). At 0, 15, 30, 60 and 120 minutes after administration, blood glucose was measured with an Accu-Chek^®^ Aviva glucose meter (Roche Diagnostics Ltd, Lewes, UK) and insulin was measured by ELISA (ChrystalChem, Chicago, IL, USA). To allow for recovery from the tests before sacrifice, juvenile and adult animals were subsequently sacrificed at 7 and 18 weeks of age, respectively. Tissues, including pancreas, liver, quadratus femoris, soleus muscle and tibiae as well as brown, subcutaneous, mesenteric and gonadal fat pads, were collected for histological assessment and gene expression analysis.

### Primary osteoblast cell culture

Primary osteoblasts (pOBs) were isolated in the calvariae of 3- to 5-day-old WT and *Enpp1^−/−^* mice as previously described (Huesa et al., 2010; Staines et al., 2014). Cells were seeded at a density of 2.5×10^4^/cm^2^ in multi-well plates, in growth medium consisting of α-MEM (Invitrogen, Paisley, UK) supplemented with 10% FBS (Invitrogen) and 1% gentamicin (Invitrogen). Cells were maintained in 95% air/5% CO_2_ at 37°C.

### Cell signalling immunoblotting

Following confluency, pOBs were cultured for 24 hours in serum-free medium containing 0.1% BSA. Cells were then either lyzed immediately or stimulated with insulin (10 nM; Sigma) or IGF-I (10 nM; Sigma) for 10 minutes before lysis. Cells were lysed in RIPA buffer (Invitrogen) containing ‘phosphatase inhibitor cocktail 2’ (Sigma) and ‘complete’ protease inhibitor cocktail (Roche) according to the manufacturers’ instructions. Immunoblotting was conducted with specific antibodies against phospho-Akt^Ser473^, total Akt, phospho-GSK3β^Ser9^, total GSK3β, phospho-Erk1/2^Thr202/Tyr204^ and total Erk1/2 (Cell Signaling, Boston, MA, USA). Immunoblotting was conducted as previously described (Zhu et al., 2011; Zhu et al., 2013) and visualized using the enhanced chemiluminescence (ECL) Western Blotting Detection System (GE Healthcare, Chalfont St Giles, UK).

### RNA extraction and qPCR

Bone and fat tissues were extracted using Qiazol (Qiagen, Valencia, CA, USA) following standard protocol procedures. All other tissues and cell cultures were extracted with the RNeasy Qiagen kit following the manufacturer’s instructions (Qiagen). RNA was quantified and reverse transcribed as previously described (Mackenzie et al., 2011; Macrae et al., 2009). All genes were analyzed with the SYBR green detection method (Roche) using the Stratagene Mx3000P real-time QPCR system (Agilent Technologies, Santa Clara, CA, USA). All gene expression data were normalized against *Atp5B* (Primer Design, Southampton, UK; sequence not disclosed) in osteoblasts, *LRP10* in adipose tissue and *Gapdh* (Primer Design; sequence not disclosed) in all other tissues. The control values were expressed as 1 to indicate a precise fold change value for each gene of interest. All primer sequences were obtained from the Harvard primer bank database (http://pga.mgh.harvard.edu/primerbank/index.html; primer sequences are available on request).

### Tissue histology and cell immunofluorescence

Dissected tissues were fixed in 4% paraformaldehyde (PFA) or 10% phosphate buffered formalin (pH 7.4) and embedded in paraffin wax. 5-μm sections were stained with hematoxylin and eosin (H&E). Fresh tissues for lipid staining were collected immediately after euthanasia, snap-frozen in precooled isopentane and stored at −80°C for less than 1 month. Cryostat sections (10 μm) were stained routinely for lipid with Oil Red O. Cells were plated onto glass coverslips, fixed in 4% PFA and permeabilized with 0.1% Triton X-100 in PBS prior to insulin receptor immunofluorescent staining following standard protocols (Huesa et al., 2010).

### Measurement of islet number and size

Serial sections were cut through each pancreas at 100-μm intervals, stained with H&E and scanned with a Nikon CoolScan V (Nikon, Surrey, UK). The total area of a stained pancreas section was measured along with the number and size of the islets in that section using ImageJ software (Wayne Rasband, National Institutes of Health, USA). At least five randomly selected serial sections were examined per pancreas and three mice were analyzed for each diet/genotype group.

### Micro-computed tomography and mechanical testing of tibiae

Tibiae were dissected and immediately frozen in PBS at −20°C pending analysis. High-resolution scans with an isotropic voxel size of 5 μm were acquired with a micro-computed tomography system (μCT, 60 kV, 0.5 mm aluminium filter, 0.6° rotation, Skyscan 1172, Brukker microCT, Kontich, Belgium). Scans were reconstructed using NRecon software (Brukker microCT). For each bone, a 1000 μm section of the metaphysis was taken for analysis of trabecular bone, using the base of the growth plate as a standard reference point. A further 1500 μm below the base of the metaphysis section, a 400 μm section of the mid-diaphysis was scanned for analysis of cortical structure. Data was analyzed with CtAn software (Brukker microCT). Cortical porosity was measured in the 2D slice-by-slice analysis. Binarized objects were identified containing fully enclosed spaces, and porosity was calculated as the area of those spaces as a percent of the total area of binarized objects. It should be noted that, for this study, total porosity is a measurement of all of the space within the cortical bone not filled by mineral, e.g. a blood vessel canal, a large osteocyte lacuna or a crack. Mechanical testing of the cortical bone was assessed by 3-point bending analysis using a Zwick materials testing machine (Zwick A.G., Ulm, Germany) with a 50 N loading cell, where the span was set at 5.5 mm and the cross-head speed was set at 1 mm/min. Data was analyzed as previously described (Huesa et al., 2011).

### Serum measurements

Immediately following euthanasia, blood was obtained from non-fasted 6-and 12-week-old male mice and serum samples prepared. Total, carboxylated (GLA), undercarboxylated (GLU13-OCN) and uncarboxylated (GLU) osteocalcin was measured as previously described (Ferron et al., 2010b), as well as markers of bone formation (P1NP; IDS, Boldon, UK) and resorption (CTx; IDS).

### Statistics

Standard comparisons between WT and *Enpp1^−/−^* mice were analyzed by unpaired Student’s *t*-test. Comparisons between genotype and diet were analyzed with two-way ANOVA. Time course experiments were analyzed with a repeated measures two-way ANOVA. Analysis was carried out using SigmaStat 12.0 (Systat Software Inc., London, UK).
